# Panel-based NGS Reveals Novel Pathogenic Mutations in Autosomal Recessive Retinitis Pigmentosa

**DOI:** 10.1038/srep19531

**Published:** 2016-01-25

**Authors:** Raquel Perez-Carro, Marta Corton, Iker Sánchez-Navarro, Olga Zurita, Noelia Sanchez-Bolivar, Rocío Sánchez-Alcudia, Stefan H. Lelieveld, Elena Aller, Miguel Angel Lopez-Martinez, Mª Isabel López-Molina, Patricia Fernandez-San Jose, Fiona Blanco-Kelly, Rosa Riveiro-Alvarez, Christian Gilissen, Jose M Millan, Almudena Avila-Fernandez, Carmen Ayuso

**Affiliations:** 1 Department of Genetics, Instituto de Investigacion Sanitaria-Fundacion Jimenez Diaz University Hospital (IIS-FJD, UAM), Madrid, Spain; 2 Centre for Biomedical Network Research on Rare Diseases (CIBERER), ISCIII, Madrid, Spain; 3 Department of Human Genetics, Radboud university medical center, 6525 GA Nijmegen, The Netherlands; 4 Grupo de Investigación en Enfermedades Neurosensoriales. Instituto de Investigación Sanitaria IIS-La Fe, Hospital Universitario La Fe, Valencia, Spain; 5 Department of Ophthalmology, IIS-Fundacion Jimenez Diaz University Hospital, Madrid, Spain

## Abstract

Retinitis pigmentosa (RP) is a group of inherited progressive retinal dystrophies (RD) characterized by photoreceptor degeneration. RP is highly heterogeneous both clinically and genetically, which complicates the identification of causative genes and mutations. Targeted next-generation sequencing (NGS) has been demonstrated to be an effective strategy for the detection of mutations in RP. In our study, an in-house gene panel comprising 75 known RP genes was used to analyze a cohort of 47 unrelated Spanish families pre-classified as autosomal recessive or isolated RP. Disease-causing mutations were found in 27 out of 47 cases achieving a mutation detection rate of 57.4%. In total, 33 pathogenic mutations were identified, 20 of which were novel mutations (60.6%). Furthermore, not only single nucleotide variations but also copy-number variations, including three large deletions in the *USH2A* and *EYS* genes, were identified. Finally seven out of 27 families, displaying mutations in the *ABCA4, RP1, RP2* and *USH2A* genes, could be genetically or clinically reclassified. These results demonstrate the potential of our panel-based NGS strategy in RP diagnosis.

Retinitis pigmentosa (RP, MIM 268000) is the most common form of inherited retinal degeneration with a prevalence of 1 in 4000 individuals^1^. RP is characterized by primary rod degeneration leading to night blindness, the development of tunnel vision and slow progressive decrease in central vision^2^. However, the disease onset, progression, retinal appearance, and final visual outcome may vary significantly among patients, even within the same family^3^. RP is also a highly heterogeneous genetic disorder; the disease can be inherited in different forms including autosomal dominant (adRP) in about 20-40% of the cases, autosomal recessive (arRP) in 30-50% or X-linked trait (xlRP) in 5-15% of the cases. Sporadic cases (sRP) account for about 40% of all diagnoses, although these percentages vary between different populations^4^,^5^. Non-Mendelian inheritance patterns such as digenic, mitochondrial or *de novo* mutations have been also reported, though these account for a minor proportion of cases^6^,^7^.

Hitherto, nearly 3100 pathogenic mutations have been reported^8^,^9^ in more than 80 genes associated with non-syndromic RP and 55 of them have been related to autosomal recessive RP (arRP) (RetNet; update Aug 2015 https://sph.uth.edu/retnet/). Although the functions of some of these genes have been extensively studied, it is difficult to establish a precise genotype-phenotype correlation, due to the fact that several genes cause distinct or partially overlapping clinical phenotypes^6^.

Genetic diagnosis of the RD has been mainly based on either the use of the specific genotyping microarrays—for which the mutation detection rate ranges from 11% to 70% depending on the RD form^10^,^11^,^12^—or on a gene-by-gene analysis by Sanger sequencing for mutation screening which is time-consuming and expensive.

Despite the difficulties, the molecular diagnosis of patients affected with RD provides them a number of benefits as it can supply an accurate prognosis of the clinical course of the disease and appropriate genetic counseling to the families. In addition, the molecular characterization of patients allows future inclusion in clinical trials based on gene therapy.

In the last few years, the development of high-throughput technologies, such as next-generation sequencing (NGS), has allowed to screen a large number of genes. Simultaneously, this technique offers a high sensitivity and efficiency making the molecular diagnosis of this heterogeneous disease easier.

NGS is currently considered the most efficient method for mutation screening. Recent studies in different populations demonstrated the great potential of NGS technologies as a diagnostic tool in the field of RD characterizing around 30-60% of cases^9^,^13^.

Herein, we report an in-house targeted gene panel developed to be an efficient and feasible tool for molecular diagnosis. Using this approach we were able to study 47 unrelated Spanish families affected by arRP. In addition, this strategy allowed us to amplify the mutational spectrum identifying not only point mutations but also copy-number variations (CNV), thereby further increasing the diagnostic yield.

## Results

**Figure 1:**
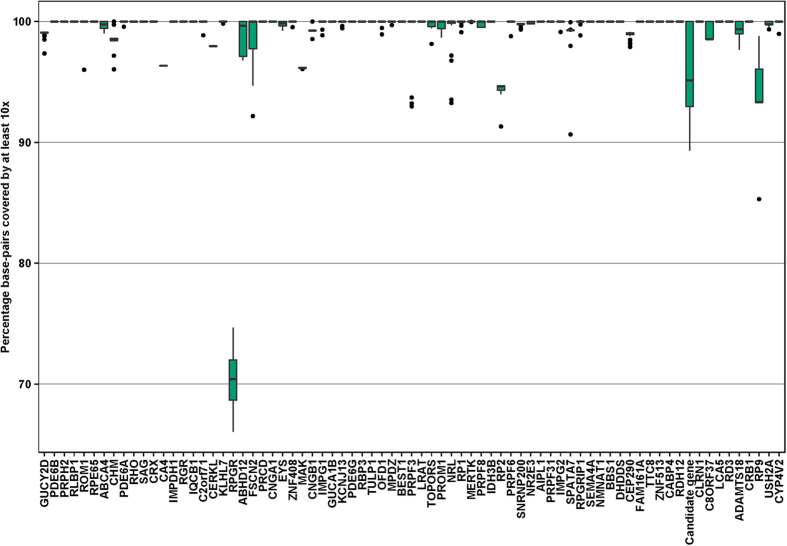
Coverage Boxplot. Overview of 75 genes and the percentage of coding bases covered by at least 10 reads. For each of the 75 genes the percentage of bases not covered by 10 or more reads is shown for the 47 samples. The green colored box indicate the first and third quantile and the black horizontal bar within the box illustrate the median (or second quantile). Measurements are considered outliers (black dots in plot) when they are: 1) less than the first quantile – 1.5xIQR or 2) greater than the third quantile +1.5 × IQR, were the IQR is the “Inter quantile range” (Third quantile – first quantile).

Next generation sequencing experiments were run on the Illumina MiSeq platform using 150 bp paired-end reads. On average 2,971,800 reads per patient were generated. On average the base-pairs of the genes present in the panel were covered by 722 times (range [92x-2911x]) and 99.1% of the base-pairs in coding regions of the 75 RD genes were covered by at least 10 reads. In addition, for 73.5% of the genes all of the base-pairs were covered by 10 reads or more. A complete overview of the coverage of each gene is shown in Fig. 1.

Because of high GC content and/or repetitive elements it was impossible to design efficient probes in seven specific regions (Supp. Table 1) of the genes: *CERKL, CA4, RP2, SNRNP200, CEP290* and *EYS*. All of these exons were additionally analyzed by Sanger sequencing though no mutations were found among the uncharacterized patients.

### Validation of the panel-based NGS

In order to verify the reliability of our NGS approach, we used a validation cohort consisting of 24 out of the total 47 patients analyzed, who had different heterozygous variants (17 mutations and 7 polymorphisms) previously identified by a genotyping microarray (Asper Biotech). Our strategy allowed us to redetect all the variants found and previously checked by Sanger sequencing giving us a sensitivity of 100% for those validation variants (Table 1).

### Genetic findings in 47 RP patients

For the 47 unrelated RP families analyzed, a total of 27 were molecularly characterized using our NGS strategy, reaching a diagnosis detection rate of 57.4% (Table 1).

After applying the filtering criteria better described in *Methods section*—which included the *in silico* prediction analysis (Table 2) and based upon the segregation analysis and the absence of those missense and splice-site novel variants in the healthy Spanish population—a total of 33 different variants were considered as causative mutations. All the variants considered as causative in the complete cohort were confirmed by Sanger sequencing therefore no false positives were found giving us a specificity of 100%.

Twenty of these causative mutations (60.6%), located in the *ABCA4, CERKL, CNGA1, EYS, PDE6A, PRCD, RDH12, RP1, RP2* and *USH2A* genes, were novel (Table 1).

Among the identified variants, 45.4% (15/33) were loss-of-function (LOF) mutations including six nonsense and nine frameshift mutations and 6% (2/33) were splice-site mutations. Approximately one-third (39.4%; 13/33) of the detected mutations were missense variants. Furthermore, three large deletions of one or more exons were found in the *USH2A* and *EYS* genes in the families RP-1706, RP-1929 and RP-2112.

In 13 out of the 27 characterized families (48.1%) one allele was previously identified by the genotyping microarray and we were able to find the second allele applying our NGS approach. For the remaining 14 characterized families (51.9%) with no previous known variation, causative mutations were found with our NGS strategy thereby establishing a molecular diagnosis. Three families with a previous mutation were not diagnosed with a second causative mutated allele whereas the fourth family with a previous mutation in *USH2A* (p.Cys759Phe) was finally diagnosed with a mutation in the *RP1* gene (RP-1772).

Based on our genetic screening, a total of seven out of the 27 cases could be reclassified. Five of them were clinically reclassified after the genetic screening. Four turned out to be atypical Usher syndrome: adult-onset mild neurosensorial hearing loss (RP-1735, RP-1979, RP-0338 and RP-0344 with mutations in *USH2A*) and one cone-rod dystrophy (CRD) (RP-1543 with mutations in *ABCA4*). The other two remained with the diagnosis of RP but turned out to be an X-linked (xlRP) and autosomal dominant retinitis pigmentosa (adRP) with mutations in *RP2* (RP-1201) and *RP1* (RP-1772) respectively (Table 1). The clinical information of all index cases of characterized families is shown in Supp. Table 2.

### CNV Analysis from High-coverage NGS Data

The high number of readouts obtained by the Illumina MiSeq Sequencing for NGS allowed us to perform a quantitative analysis.

**Figure 2:**
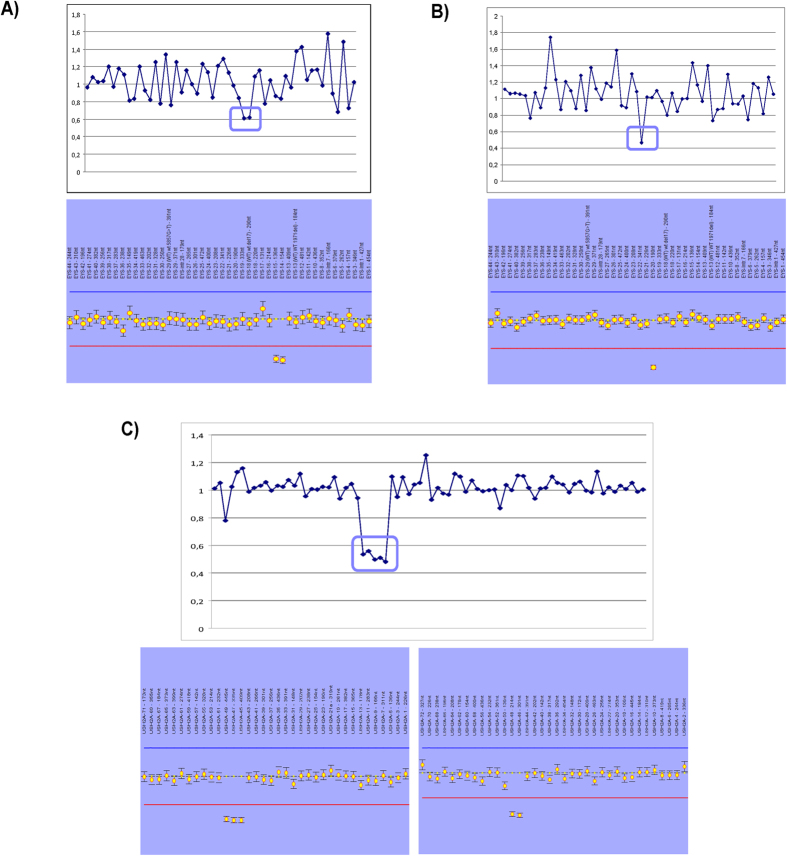
CNVs analysis and MLPA profile for the three large rearrangements. Ratios below 0.7 were considered deletions; those above 1.2 were considered to be duplications. (**A**) Deletion of exons 13 and 14 identified in the *EYS* gene in the RP-1929 family. (**B**) Deletion of exon 19 identified in the *EYS* gene in the RP-1706 family. (**C**) Gross deletion from the exon 45 to 49 in *USH2A* (RP-2112).

Only targeted regions covered by at least 100X, in order to avoid false positive calls, were selected for the CNV analysis. After the CNV study carried out in our cohort, five large copy-number changes in a heterozygous state, of which two deletions in the *EYS* gene, one deletion in *USH2A* and two different duplications in the *CEP290* gene, were detected (Supp. Table 3). The two deletions identified in the *EYS* gene comprised exons 13-14 (previously reported) and exon 19 (firstly reported in this study), whereas a deletion from the exon 45 to 49 previously reported by *Baux et al.*^14^ was detected in *USH2A* (Table 1). All copy number variants were subsequently validated by MLPA (Fig. 2). To check the *CEP290* duplications in the RP-0830 family, a customized-aCGH previously designed in our group for another purpose^15^ including several genes was used but the duplications could not be confirmed (data not shown).

### Uncertain variants and unsolved cases

Novel missense variants predicted as pathogenic were found. However their high frequency in Spanish population (SPV database) leads us to classify them as benign (Supp Table 4).

For the family RP-1874 only one pathogenic allele, a non-canonical splice site mutation in *CNGB1* (c.2634 + 6G > A) was identified (Table 3). The index case of the RP-1747 family carried two potentially pathogenic heterozygous mutations in two different genes, *EYS* (p.Tyr2822Cys) and *USH2A* (p.Gly1871Asp) however no second allele was found (Table 3). Finally, in 17 out of 47 families (36.1%) no candidate variants were found.

## Discussion

Recently, the NGS has been proved to be very helpful for carrying out genetic studies in heterogeneous diseases such as Retinitis Pigmentosa^13^,^16^. In our study, a targeted sequence capture to detect novel pathogenic variants in 75 known RP genes in 47 arRP Spanish families was performed.

Twenty-seven out of 47 families were solved (detection rate of 57.4%) identifying 33 different mutations. From them, 60.6% (20/33) were novel mutations affecting the *ABCA4, CERKL, CNGA1, EYS, PDE6A, PRCD, RDH12, RP1, RP2* and *USH2A* genes. All known variants used as positive controls were redetected by this technology demonstrating the high specificity and sensitivity of our approach.

Unfortunately, for those cases with only one pathogenic allele or with two heterozygous mutations in different genes, cosegregation analysis could not be performed or provided results were inconclusive. Further transcriptional profile studies may clarify the effect of the non-canonical splice-site mutation found in the *CNGB1* gene. Some authors have claimed to have found evidence of digenic inheritance when they detect two clear mutations in two different genes^17^. However, as has been previously reported, this event is rarely demonstrated, since the genetic load of mutations in genes responsible for RP is very high as has been previously reported^18^. In the family RP-1747 that carried two likely pathogenic variants in *USH2A* and *EYS* we cannot exclude the presence of a second pathogenic allele in deep intronic or other genomic regions that are not systematically analyzed, and a third mutation is found by chance in the second gene. Alternatively it could be that the gene responsible for the disease had not yet been identified, and the possible modifier effect of the mutations found in the *EYS* and *USH2A* genes remain to be demonstrated.

According to the molecular results, seven out of 27 families were both clinically and genetically reclassified. In order to provide an accurate genetic counseling, disease prognosis and follow up to the families, their reclassification is crucial. In addition, it allows to develop new personalized therapies for these families. Since mutations in *USH2A* cause both non-syndromic RP and Usher syndrom^19^,^20^, the presence of the mutations in this gene in the families requires the hearing follow up for these patients. The RP-1543 family with mutations in *ABCA4* was previously classified as arRP however, the presence of these two mutations arguing in favour of a cone-rod dystrophy allowed after a careful clinical evaluation its reclassification^21^. It is well known that at advance stages of the disease it is difficult to establish a precise clinical diagnosis. The family RP-1201 presented a mutation in the *RP2* X-linked gene changing dramatically the genetic counseling for the family. In addition previous studies have demonstrated that around 15% of males with isolated RP carry mutations in X-linked retinal degeneration genes^22^.

Lastly the RP-1772 family, which carried a heterozygous *RP1* mutation, was re-classified as an autosomal dominant RP. Although mutations in the *RP1* gene have been described in both, autosomal dominant and recessive RP forms^23^, type and location of the mutation and phenotype can help in the genetic classification. The novel frameshift mutation was located in the protein domain where mutations were usually found causing autosomal dominant RP. Also, the phenotype of the patients argues in favour of this finding due to the recessive mutations in the *RP1* gene cause an early onset RP^24^. Although there was not antecedents of retinal degeneration in the family, we could confirm the frameshift mutation also in a non-affected sibling supporting the existence of the incomplete penetrance previously reported in this gene^25^. Thus, the identification of the genetic defect becomes increasingly relevant, especially when gene-specific therapeutic approaches are becoming more promising according to the results of the ongoing clinical trials^26^.

It is important to be aware of the NGS limitations. The detection of some types of mutations could be missed as trinucleotide repeats, small insertions/deletions or CNV variations^27^. In our design, for *RPGR* only 70% of the whole gene was covered by at least 10X. Due to the highly repetitive sequences and purine-rich regions in open reading frame 15 (ORF15)─ that is the last exon of the *RPGR* gene in which nearly 60% of disease-causing *RPGR* mutations are located─is difficult to capture using conventional NGS technologies. However, the rest of the gene was greatly covered. To date other alternatives have been successfully performed to sequence ORF15 in those cases in which attempts at characterization by NGS had failed^28^.

The high coverage obtained with our panel-based NGS approach allows us to perform a CNV analysis in our cohort providing the genetic diagnosis for three families (3/47). Although the CNVs results could not be reliable when the coverage fell below 100X, this analysis should be routinely applied in targeted sequencing.

Regardless of the inheritance pattern, in well-characterized cases, the panel-based targeted sequencing is superior to other methods in both time and cost, which makes it an optimal approach. Compared with other technologies used in the past such as genotyping microarrays^12^, NGS has been reported to identify the genetic cause in 19% to 50% of arRP cases (and 50% to 82% of RP cases in general)^10^,^29^, which is significantly higher than the reported 11-12% for microarray screening in Spanish population.

Our results extend not only the mutational spectrum for arRP but also the population specific variants frequently found in our Spanish database and facilitates further interpretation of NGS panel on RD patients. Our cohort was selected after a previous screening with a genotyping microarray and 11 out of the 17 families with a previous mutated allele had a mutation in the *USH2A* gene enriching the percentage of *USH2A* mutations. Therefore, our results are not representative of the frequency and distribution of the genes in the Spanish population.

The obtained detection rate of 57.4% supports the application of this targeted NGS strategy as an effective tool for the diagnosis of RP patients^30^ because our approach can handle the heterogeneity of the disease. Further improvements in NGS technologies and the discovery of novel genes involved in the disease will likely improve the molecular diagnosis of RP.

## Methods

### Patients recruitment

Patients diagnosed with RP were recruited from the Biobank of the Fundación Jiménez Díaz Hospital (Madrid, Spain). Diagnostic criteria for RP included night blindness and/or peripheral visual loss and poor visual acuity in advanced stages of the disease. Ophthalmic examinations including electrorretinogram (ERG), fundus, visual field test and best-corrected visual acuity (BCVA) measurements were performed on each index case.

Peripheral blood samples of index cases and their family members were collected in EDTA tubes. DNA was extracted from peripheral blood leukocytes with an automated DNA extractor (model BioRobotEZ1; Qiagen, Hilden, Germany) following the manufacturer’s instructions.

A total of 47 unrelated Spanish families with arRP or sporadic RP (sRP), previously screened for known mutations with a specific arRP genotyping microarray (AsperBiotech, Tartu, Estonia) were selected. The cohort included 24 patients with a previously identified variation (mutation or polymorphism) used as validation controls and 23 without any previous genetic alteration. Informed consents were obtained from all patients and family members involved in the study. All procedures were reviewed and approved by the Ethics Committee of the hospital and adhered to the tenets of the Declaration of Helsinki and further reviews (Fortaleza, 2013).

Two hundred and sixty-seven in-house whole exomes from Spanish individuals (Spanish Population Variations Database available in the public domain at http://bioinfo.cipf.es/apps-beta/spv/1.0.1/ and created by Bioinformatic Platform for Rare Diseases CIBERER-BIER, http://www.ciberer.es/bier/) without RP family history of RP were used as controls to evaluate the frequency of the variations found in this study.

### Capture panel design

A customized Haloplex panel was designed to capture 75 RD genes (74 previously known and one candidate gene) of which 41 were linked to arRP (RetNet; update Sept 2015 https://sph.uth.edu/retnet/).

The design was performed as previously described^30^. The capture sequences included all coding exons and 20 bp of their flanking 5′ and 3′ intronic regions. Additionally, four known deep-intronic mutations, involving the genes *CEP290*, *OFD1*, *PRPF31* and *USH2A* were covered. In total 1127 regions were targeted comprising 352 Kb of target sequence. The final design covered 99.1% of the requested target regions.

### Targeted NGS approach

The HaloPlex enrichment system is based on restriction enzyme digestion of genomic DNA followed by hybridization of customized probes to capture regions of interest, which are subsequently amplified by PCR.

The sequence capture was performed following the manufacturer’s protocol (version D3, December 2012) for Illumina Sequencing protocol (Agilent Technologies Inc., 131 Santa Clara, CA, USA) with minor revisions as previously described^30^. Capture libraries were sequenced using the MiSeq v2 reagents kit (Illumina, San Diego, California, USA) on 8 runs in an Illumina MiSeq as 150 bp paired-end reads, following the manufacturer’s protocol.

The primary analysis, including base calling and quality scoring, was performed by the Illumina RTA software application.

### Data analysis

Results were analyzed with two different custom pipelines: DNAnexus analysis (www.dnanexus.com) and BaseSpace BWA Enrichment v2.1 analysis (Illumina, San Diego, California, USA). For both, sequence reads from Illumina MiSeq instrument were mapped to the hg19/GRCh37 human reference genome. The alignment and variant calling were performed using Burrows-Wheeler Aligner (BWA)-MEM^31^ and Genome Analysis Toolkit (GATK) respectively, with default parameters depending on the pipeline used. The CalculateHsMetrics tool in Picard was used to produce the reads and coverage statistics (www.picard.sourceforge.net).

Single base pair coverage for the 75 genes was calculated based on the BAM files with the use of the *coverage* function of BEDtools (v2.19.1)^32^. The coverage on base-pair resolution was computed for the protein coding regions (hg19 assembly) of the NCBI RefSeq database (Release 70)^33^. The RefSeq transcripts were downloaded from the UCSC genome browser and converted to bed format by a custom Java program. For a total of 185,751 bases the coverage was determined.

The output VCF files were annotated in order to identify and classify the disease-relevant variants using the GATK Variant Annotator and ENSEMBL Variant Effect Predictor v72 for the samples analyzed with DNAnexus and the VariantStudio Variant Analysis Software (Illumina, San Diego, California, USA) for the samples analyzed with the BaseSpace BWA Enrichment v2.1.

All identified variants were annotated according to the guidelines published by the Human Genome Variation Society (www.hgvs.org/mutnomen) and were checked in the dbSNP v132 and Human Gene Mutation Data (HGMD^®^ Professional) databases.

### Variant prioritization and pathogenicity assesment

Identified variants were filtered by applying the following prioritization criteri^4^,^30^:

- (1) Variants in the coding sequence excluding intergenic, 5′ and 3′ untranslated regions or deep intronic variants were selected.
- (2) Variants were filtered by minor allele frequency (MAF) ≤ 0.005 or without MAF value in the 1000 Genomes Project, Exome Variant Server (EVS) and Exome Aggregation Consortium (ExAC).
- (3) Variants were checked in dbSNP132

- Variants with *rs* number and present in Human Gene Mutation Data (HGMD^®^ Professional) and Leiden Open Variation Database (LOVD), were considered as known pathogenic mutations after all the references were checked.
- Variants without *rs* number were considered as novel rare variants.

- (4) Nonsense, frameshift and canonical splice-site variants were considered to be pathogenic.
- (5) Missense or non-canonical splice-site novel rare variants were checked against the Spanish Population Variation Database (BIER), including 267 exomes from healthy individuals. If a given variant was not present, the *in silico* prediction was performed.
- (6) To assess the pathogenesis of the missense variants, four different predictive software programs were used including: i) Sorting Intolerant from Tolerant (SIFT; sift.jcvi.org), ii) Polymorphism Phenotyping v2 (Polyphen-2; genetics.bwh.harvard.edu/pph2), iii) Align GVGD (agvgd.iarc.fr/agvgd_input.php) and iv) Mutation Taster (www.mutationtaster.org). Variants detected in potential splice-sites were analyzed by: i) Human Splice Finder (HSF; www.umd.be/HSF), ii) Analyzer Splice Tool (AST; ibis.tau.ac.il/ssat/SpliceSiteFrame.htm), iii) Berkeley Drosophila Genome Project (BDGP; www.fruitfly.org/seq_tools/splice), iv) NetGene 2 (www.cbs.dtu.dk/services/NetGene2) and v) ESE Finder 3.0 (http://rulai.cshl.edu/cgi-bin/tools/ESE3/esefinder.cgi?process=home). Those variants predicted as damaging by at least two different prediction softwares were initially considered potentially pathogenic.

For those samples with a previous pathogenic variant a manual search for a second variant allele in the corresponding gene was performed directly. When a second pathogenic allele was not found we applied the previous filtering criteria in order to find other RP genes. Variants were further prioritized according to the inheritance pattern.

### Data validation and segregation analysis

A total of 24 patients carrying a previous known variant (17 mutations and 7 polymorphism) were tested as validation controls to verify the reliability of our custom NGS strategy.

Sanger sequencing was performed to confirm all the deleterious mutations and potentially pathogenic variants and to segregate them in the families. Bidirectional automatic sequencing was performed using 20-mer oligonucleotide primer pairs designed by ExonPrimer software (UCSC). Primer sequences and annealing temperatures are available from the authors on request. The PCR products were enzymatically purified with ExoSAP-it (USB, Affymetrix) and sequenced using BigDye^®^ Terminator v1.1 Cycle Sequencing Kit (Life technologies). The PCR products were resolved on an automated sequencer (ABI 3130xl Genetic Analyzer, Applied Biosystems). The results were analyzed by Staden Package software version 2.0.0 b10 (available at: staden.sourceforge.net; accessed September, 2014) by assembling and visualizing the aligned sequences compared with reference sequence (UCSC Genome Browser).

### Copy-number variation analysis

Due to the very high expected coverage for all target regions sequenced with the Illumina MiSeq system (>90% base-pairs covered by 100×), a copy-number variation (CNV) analysis was performed. The coverage of each target region was normalized and compared against normalized data of the rest of the samples of the same run to obtain the ratio relative coverage^34^. Only those cases with a heterozygous pathogenic single-nucleotide mutation in a specific gene were analyzed. If the ratio fell below 0.7 it was considered a deletion and if it rose above 1.2 it was considered a duplication. In addition only those regions with ≥ 100× were selected. Following these criteria the detected CNVs were validated by multiplex ligation-dependent probe amplification (MLPA) for the affected genes. For the *EYS* gene, the SALSA MLPA probemix P328 was used. To confirm the *USH2A* CNV the SALSA MLPA probemixes P361 and P362 were utilized (MRC-Holland, Amsterdam, The Netherlands). The MLPA reaction was performed following the manufacturer’s instructions. The MLPA results were analyzed with the Coffalyser.Net software (MRC-Holland, Amsterdam, The Netherlands).

The validation of the putative CNVs found in *CEP290*, in which commercial MLPA was not available, was performed by a customized comparative genomic hybridization array (aCGH).

### Comparative genomic hybridization array (aCGH)

The customized aCGH 8 × 60 k Agilent SurePrint G3 CGH was designed using the Agilent eArray website (https://earray.chem.agilent.com/earray/) with an average distribution of 1 probe per 150 pb.

The processing was performed according to manufacturer’s recommendations as previously described^15^.

Results were analyzed by Agilent CytoGenomics software v.2.7 using default analysis method – CGH v2 with the ADM-2 aberration algorithm.

## Additional Information

**How to cite this article**: Perez-Carro, R. *et al.* Panel-based NGS Reveals Novel Pathogenic Mutations in Autosomal Recessive Retinitis Pigmentosa. *Sci. Rep.* **6**, 19531; doi: 10.1038/srep19531 (2016).

## Supplementary Material

Supplementary Table 1

Supplementary Table 2

Supplementary Table 3

Supplementary Table 4

## Figures and Tables

**Table 1:** Causative mutations identified in 27 characterized autosomal recesive retinitis pigmentosa families.

**Family**	**Gene**	**Allele 1**				**Allele 2**				
		Nucleotidechange	**Protein Change**	**Methods**	**Reference**	**Nucleotide change**	**Protein Change**	**Methods**	**Reference**	**Segregation**
RP-1147	*CNGA1*	c.94C > T	p.Arg32*	aC	Paloma (2002)	c.830G > A	p.Arg277Gln	H	This study	Yes
RP-2066	*PDE6A*	c.1705C > A	p.Gln569Lys	aC	Dryja (1999)	c.1620 + 1G > T	splicing defect	H	This study	NA
RP-2114	*RDH12*	c.278T > C	p.Leu93Pro	aC	Avila-Fernandez (2010)	c.210–211insC	p.Arg71Glnfs*12	H	This study	NA
RP-1319	*USH2A*	c.2276G > T	p.Cys759Phe	aC	Rivolta (2000)	c.920–923dupGCCA	p.His308Glnfs*16	H	Weston (2000)	NA
RP-1412	*USH2A*	c.2276G > T	p.Cys759Phe	aC	Rivolta (2000)	c.1214delA	p.Asn405Ilefs*3	H	Schwartz (2005)	Yes
RP-1646	*USH2A*	c.2276G > T	p.Cys759Phe	aC	Rivolta (2000)	c.12575G > A	p.Arg4192His	H	Avila-Fernandez (2010)	Yes
RP-1695	*USH2A*	c.2276G > T	p.Cys759Phe	aC	Rivolta (2000)	c.9799T > C	p.Cys3267Arg	H	Aller (2006)	NA
RP-1735	*USH2A*	c.2276G > T	p.Cys759Phe	aC	Rivolta (2000)	c.12153–12175delAATTTTAAGCCCTTGGACTCTGA	p.Glu4051Aspfs*40	H	This study	NA
RP-1802	*USH2A*	c.2276G > T	p.Cys759Phe	aC	Rivolta (2000)	c.920–923dupGCCA	p.His308Glnfs*16	H	Weston (2000)	Yes
RP-1976	*USH2A*	c.2276G > T	p.Cys759Phe	aC	Rivolta (2000)	c.8254G > A	p.Gly2752Arg	H	Nakanishi (2009)	NA
RP-1979	*USH2A*	c.2276G > T	p.Cys759Phe	aC	Rivolta (2000)	c.1606T > C	p.Cys536Arg	H	Dreyer (2000)	Yes
RP-2112	*USH2A*	c.2276G > T	p.Cys759Phe	aC	Rivolta (2000)	Deletion Ex.45-49	–	H	Baux (2014)	NA
RP-2113	*USH2A*	c.2276G > T	p.Cys759Phe	aC	Rivolta (2000)	c.5462A > G	p.Lys1821Arg	H	This study	Yes
RP-1543	*ABCA4*	c.4234C > T	p.Gln1412*	H	Maugeri (1999)	c.5917delG	p.Val1973*	H	Rivera (2000)	NA
RP-1056	*CERKL*	c.847C > T	p.Arg283*	H	Tuson (2004)	c.664C > T	p.Gln222*	H	This study	Yes
RP-1998	*CNGB1*	c.2957A > T	p.Asn986Ile	H	Simpson (2011)	c.2957A > T	p.Asn986Ile	H	Simpson (2011)	Yes
RP-1706	*EYS*	c.2826–2827delAT	p.Val944Glyfs*9	H	This study	Deletion Ex.19	–	H	This study	Yes
RP-1929	*EYS*	c.9142dupA	p.Arg3048Lysfs*9	H	This study	Deletion Ex.13-14	–	H	Pieras (2011)	NA
RP-1142	*GUCY2D*	c.1762C > T	p.Arg588Trp	H	Stone (2007)	c.1762C > T	p.Arg588Trp	H	Stone (2007)	Yes
RP-0372	*PDE6A*	c.1957C > T	p.Arg653*	H	This study	c.1957C > T	p.Arg653*	H	This study	Yes
RP-0040	*PRCD*	c.74 + 1G > A	splicing defect	H	This study	c.74 + 1G > A	splicing defect	H	This study	Yes
RP-1772	*RP1*	c.2431delA	p.Ser812Valfs*36	H	This study					Yes
RP-1988	*RP1*	c.400–401insGC	p.His136Argfs*8	H	This study	c.400–401insGC	p.His136Argfs*8	H	This study	Yes
RP-1201	*RP2*	c.708C > G	p.Cys236Trp	H	This study					Yes
RP-0338	*USH2A*	c.9433C > T	p.Leu3145Phe	H	This study	c.9433C > T	p.Leu3145Phe	H	This study	Yes
RP-0344	*USH2A*	c.8693A > C	p.Tyr2898Ser	H	de Castro-Miró (2014)	c.10008C > A	p.Cys3336*	H	This study	Yes
RP-0456	*USH2A*	c.2276G > T	p.Cys759Phe	H	Rivolta (2000)	c.10709G > T	p.Cys3570Phe	H	This study	NA

Abbreviations: arRP Chip (aC); Haloplex (H). NA: No additional family members available.

**Table 2:** Pathogenicity assesment of novel rare missense and splicing variants.

**Missense Variants**											
**Family**	**Gene**	**cDNA**	**Protein**	**SIFT**	**Polyphen**	**Align GVGD**	**Mut.Taster**	**phyloP**	**SPV**	**EVS**	**ExAC**
RP-1147	*CNGA1*	c.1037G > A	p.Arg346Gln	D(0)	Pos. D(0.852)	Class C35	D	5.39	–	0.0001	0.00001
RP-1201	*RP2*	c.708C > G	p.Cys236Trp	D(0)	Pr. D(0.997)	Class C65	D	-0.149	–	–	–
RP-0456	*USH2A*	c.10709G > T	p.Cys3570Phe	D(0)	Pr. D(0.996)	Class C65	D	5.21	–	–	–
RP-0338	*USH2A*	c.9433C > T	p.Leu3145Phe	D(0.02)	Pos. D(0.598)	Class C15	P	0.70	–	0.0001	–
RP-2113	*USH2A*	c.5462A > G	p.Lys1821Arg	T(0.1)	B(0.096)	Class C25	P	2.00	–	–	–
**Splice Variants**											
**Family**	**Gene**	**cDNA**	**Protein**	**HSF**	**BDGP**	**AST**	**NetGene 2**	**ESEFinder**	**SPV**	**EVS**	**ExAC**
RP-2066	*PDE6A*	c.1620 + 1G > T	splicing defect	95.1/-	0.99/-	86.85/69.73	0.82/-	loss SF2/ASF	–	–	0.00001
RP-0040	*PRCD*	c.74 + 1G > A	splicing defect	79.8/-	0.45/-	67.08/49.96	0.7/-	loss SF2/ASF	–	–	0.00002

Abbreviations: SIFT ( D: deleterious; T: tolerated); Polyphen (Pos.D: possibly damaging; Pr.D: probably damaging; B: benign); Align GVGD (Class C15 less likely pathogenic and Class C65 most likely pathogenic); Mutation taster (D: disease causing; P: polymorphism). PhyloP (From negative values to 1: fast-evolving sites and positive values above 1: conserved sites). Splicing prediction software: wild-type/mutant score. Human Splice finder (HSF), Berkeley Drosophila Genome Project (BDGP), Analyzer Splice Tool (AST). – means the depletion of the 5’ splice site. SPV: Spanish Population Variation Database. EVS: Exome Variant Server. ExAC: Exome Aggregation Consortium.

**Table 3:** Unsolved families carrying one pathogenic allele.

**Splicing**													
**Family**	**Gene**	**Nucleotide change**	**Protein Change**	**HSF**	**BDGP**	**NetGene 2**	**ESEFinder**	**1000G**	**EVS**	**ExAC**	**SPV**	**Reference**	
RP-1874	*CNGB1*	c.2634 + 6G > A	splicing defect	New cryptic acceptor site	New cryptic acceptor site	New cryptic acceptor site	loss SRp40	–	–	0.000008	–	This study	
**Missense**													
**Family**	**Gene**	**Nucleotide change**	**Protein Change**	**SIFT**	**Polyphen**	**Align GVGD**	**Mut.Taster**	**phyloP**	**1000G**	**EVS**	**ExAC**	**SPV**	**Reference**
RP-1747	*EYS*	c.8465A > G	p.Tyr2822Cys	Not scored	Pr.D(0.997)	Class C0	P	1.23	–	–	–	–	This study
RP-1747	*USH2A*	c.5612G > A	p.Gly1871Asp	D(0)	Pr.D(0.995)	Class C65	D	2.81	0.0016	–	0.0003	–	This study

Abbreviations: SIFT ( D: deleterious; T: tolerated); Polyphen (Pos.D: possibly damaging; Pr.D: probably damaging; B: benign); Mutation taster (D: disease causing; P: polymorphism). Splicing prediction software: wild-type/mutant scores. Human Splice finder (HSF), Berkeley Drosophila Genome Project (BDGP), Analyzer Splice Tool (AST). EVS: Exome Variant Server. ExAC: Exome Aggregation Consortium. SPV: Spanish Population Variation Database.
