# Corrigendum: Panel-based NGS Reveals Novel Pathogenic Mutations in Autosomal Recessive Retinitis Pigmentosa

**DOI:** 10.1038/srep24843

**Published:** 2016-04-22

**Authors:** Raquel Perez-Carro, Marta Corton, Iker Sánchez-Navarro, Olga Zurita, Noelia Sanchez-Bolivar, Rocío Sánchez-Alcudia, Stefan H. Lelieveld, Elena Aller, Miguel Angel Lopez-Martinez, Mª Isabel López-Molina, Patricia Fernandez-San Jose, Fiona Blanco-Kelly, Rosa Riveiro-Alvarez, Christian Gilissen, Jose M Millan, Almudena Avila-Fernandez, Carmen Ayuso

Scientific Reports 6: Article number: 1953110.1038/srep19531; published online: 01252016; updated: 04222016

This Article contains errors.

In Table 1, for Family RP-1929 the nucleotide change ‘c.9079dupA’ and protein change ‘p.Arg3027Lysfs*9’ were incorrectly given as ‘c.9142dupA’ and ‘p.Arg3048Lysfs*9’ respectively.

In Table 2, for Family RP-1147 the cDNA ‘c.830G > A’ and protein ‘p.Arg277Gln’ were incorrectly given as ‘c.1037G > A’ and ‘p.Arg346Gln’ respectively.

