# Supplementary material for: Panel-based NGS Reveals Novel Pathogenic Mutations in Autosomal Recessive Retinitis Pigmentosa
Source: Sci Rep. 2016 Jan 25;6:19531. doi: 10.1038/srep19531 (PMC4726392; doi:10.1038/srep19531)
Supplement: Supplementary Table 1 [file srep19531-s1.pdf]

Panel-based NGS Reveals Novel Pathogenic Mutations in Autosomal Recessive Retinitis Pigmentosa

Raquel Perez-Carro[1,2], Marta Corton[1,2], Iker Sánchez-Navarro[1,2], Olga Zurita[1,2], Noelia Sanchez-Bolivar[1,2], Rocío Sánchez-Alcudia[1,2], Stefan H. Lelieveld[3], Elena Aller[2,4], Miguel Angel Lopez-Martinez[1,2], Mª Isabel López-Molina[5], Patricia Fernandez-San Jose[1,2], Fiona Blanco-Kelly[1,2], Rosa Riveiro-Alvarez[1,2], Christian Gilissen[3], Jose M Millan[2,4], Almudena Avila-Fernandez[1,2,6], Carmen Ayuso* [1,2,6].

Supplementary Table 1. Regions not covered in our RD panel.

Chr	Start position	End position	Gene	Exon
2	96967395	96967407	*SNRNP200*	4
2	182468774	182468826	*CERKL*	2
6	64497950	64498012	*EYS*	39
12	88471664	88471677	*CEP290*	40
12	88510789	88510829	*CEP290*	18
17	58232683	58232718	*CA4*	2
X	46713082	46713137	*RP2*	2
