# Supplementary material for: Panel-based NGS Reveals Novel Pathogenic Mutations in Autosomal Recessive Retinitis Pigmentosa
Source: Sci Rep. 2016 Jan 25;6:19531. doi: 10.1038/srep19531 (PMC4726392; doi:10.1038/srep19531)
Supplement: Supplementary Table 2 [file srep19531-s2.pdf]

**Panel-based NGS Reveals Novel Pathogenic Mutations in Autosomal Recessive Retinitis Pigmentosa**

Raquel Perez-Carro[1,2], Marta Corton[1,2], Iker Sánchez-Navarro[1,2], Olga Zurita[1,2], Noelia Sanchez-Bolivar[1,2], Rocío Sánchez-Alcudia[1,2], Stefan H. Lelieveld[3], Elena Aller[2,4], Miguel Angel Lopez-Martinez[1,2], Mª Isabel López-Molina[5], Patricia Fernandez-San Jose[1,2], Fiona Blanco-Kelly[1,2], Rosa Riveiro-Alvarez[1,2], Christian Gilissen[3], Jose M Millan[2,4], Almudena Avila-Fernandez[1,2,6], Carmen Ayuso* [1,2,6].

**Supplementary Table 2**. Clinical findings identified in the index cases of the characterized families.

| Family | Symptoms and course | Fundus | ERG | Other features |
|---|---|---|---|---|
| RP-1147 | NB (16y), VF constriction (30y) and VA diminished (30y) | Bone spicule pigmentation | ND | |
| RP-2066 | NB (6y), loss of VF (6y) and VA diminished (10y) | Bone spicule pigmentation in mid periphery | ND | |
| RP-2114 | NB (3y), loss of VF (3y) and VA diminished (4y) | Attenuated vessels and bone spicule pigmentation. | NR | Photofobia |
| RP-1319 | NB (20y), VF constriction (22y) and loss of VA (28y) | Bone spicule pigmentation and macular edema. | ND | |
| RP-1412 | NB (18y), VF constriction (30y) and loss of VA (38y) | ND | ND | |
| RP-1646 | NB (48y), VF constriction (48y) and loss of VA (53y) | Bone spicule pigmentation | ND | |
| RP-1695 | NB (7y), loss of VF (13y) and VA diminished (13y) | ND | NR | Photofobia |
| RP-1735 | NB (30y), loss of VF (30y) and VA diminished (43y) | Pale optic disc. Bone spicule pigmentation and attenuated vessels. | Reduced a-wave and b-wave amplitudes | Bilateral sensorineural hearing loss |
| RP-1802 | NB (48y), VF constriction (48y) and loss of VA (53y) | Bone spicule pigmentation | ND | Posterior subcapsular cataract (45y) |
| RP-1976 | NB (40y), VF constriction (50y) and loss of VA (56y) | Pale optic disc. Bone spicule pigmentation and attenuated vessels. | NR | Posterior subcapsular cataract (66y) Photofobia |
| RP-1979 | NB (30y), VF constriction (30y) and loss of VA (33y) | Pale optic disc. Bone spicule pigmentation and attenuated vessels. | NR | Posterior subcapsular cataract (49y). Bilateral sensorineural hearing loss |
| RP-2112 | NB (30y), loss of VF (36y) and VA diminished (39y) | Bone spicule pigmentation | NR | |
| RP-2113 | NB (35y), loss of VF (50y) and VA diminished (55y) | Pale optic disc. Bone spicule pigmentation | ND | Posterior subcapsular cataract (62y). Photofobia |
| RP-1543 | NB (20y), VF constriction (23y) and loss of VA (8y) | Pale optic disc. Bone spicule pigmentation | NR | Nystagmus |
| RP-1056 | NB (21y), loss of VF (28y) and VA diminished (30y) | ND | ND | Posterior subcapsular cataract (38y) |
| RP-1998 | NB (12y), loss of VF (12y) and VA diminished (27y) | Attenuated vessels and initial pigment deposits | Reduced a-wave and b-wave amplitudes | |
| RP-1706 | NB (10y), VF constriction (6y) and loss of VA (15y) | Pale optic disc. Bone spicule pigmentation and attenuated vessels. | NR | Posterior subcapsular cataract (42y) |
| RP-1929 | NB (10y), VF constriction (10y) and loss of VA (10y) | Pale optic disc. Bone spicule pigmentation | ND | Posterior subcapsular cataract (70y) |
| RP-1142 | NB (<10y), loss of VF (<10y) and VA diminished (<10y) | Pale optic disc. Bone spicule pigmentation and attenuated vessels. | NR | Posterior subcapsular cataract (70y). Dyschromatopsia |
| RP-0372 | NB (5y), loss of VF (5y) and VA diminished (5y) | Bone spicule pigmentation and attenuated vessels. | Reduced a-wave and b-wave amplitudes | |
| RP-0040 | NB (25y), VF constriction (25y) and loss of VA (28y) | Pale optic disc. Bone spicule pigmentation and attenuated vessels. | NR | Posterior subcapsular cataract (34y) |
| RP-1772 | NB (20y), VF constriction (35y) and loss of VA (45y) | Bone spicule pigmentation and attenuated vessels. | Reduced a-wave and b-wave amplitudes | Photofobia |
| RP-1988 | NB (6y), VF constriction (6y) and loss of VA (6y) | Bone spicule pigmentation and attenuated vessels. | ND | Photofobia. Dyschromatopsia |
| RP-1201 | NB (16y), loss of VF (20y) and VA diminished (26y) | ND | ND | |
| RP-0338 | NB (35y), loss of VF (35y) and VA diminished (35y) | Bone spicule pigmentation in mid periphery and attenuated vessels | NR | Bilateral sensorineural hearing loss |
| RP-0344 | NB (21y), VF constriction (28y) | Pale optic disc. Bone spicule pigmentation and attenuated vessels. | NR | Photofobia. Bilateral sensorineural hearing loss |
| RP-0456 | NB (38y), VF constriction (40y) and loss of VA (42y) | ND | ND | Posterior subcapsular cataract (40y) |

Abbreviations: NB(night blindness); VF(VF); VA(visual acuity);ERG(electroretinogram); NR( non-recordable);ND(no data).
