# Supplementary material for: Panel-based NGS Reveals Novel Pathogenic Mutations in Autosomal Recessive Retinitis Pigmentosa
Source: Sci Rep. 2016 Jan 25;6:19531. doi: 10.1038/srep19531 (PMC4726392; doi:10.1038/srep19531)
Supplement: Supplementary Table 3 [file srep19531-s3.pdf]

Panel-based NGS Reveals Novel Pathogenic Mutations in Autosomal Recessive Retinitis Pigmentosa

Raquel Perez-Carro[1,2], Marta Corton[1,2], Iker Sánchez-Navarro[1,2], Olga Zurita[1,2], Noelia Sanchez-Bolivar[1,2], Rocío Sánchez-Alcudia[1,2], Stefan H. Lelieveld[3], Elena Aller[2,4], Miguel Angel Lopez-Martinez[1,2], Mª Isabel López-Molina[5], Patricia Fernandez-San Jose[1,2], Fiona Blanco-Kelly[1,2], Rosa Riveiro-Alvarez[1,2], Christian Gilissen[3], Jose M Millan[2,4], Almudena Avila-Fernandez[1,2,6], Carmen Ayuso* [1,2,6].

Supplementary Table 3. Targeted regions identified with a copy number variations. If the ratio fell below 0.7 it was considered a deletion and if rose above 1.2 was considered as duplication.

Family	chr	Start position	End position	Gene	Exon	Ratio	Validation
RP-1706	6	65596359	65596964	*EYS*	19	0.46	MLPA (+)
RP-1929	6	65707432	65707849	*EYS*	14	0.61	MLPA (+)
	6	65767362	65767710	*EYS*	13	0.62	MLPA (+)
RP-2112	1	215986857	215987433	*USH2A*	49	0.51	MLPA (+)
	1	215990172	215990713	*USH2A*	48	0.53	MLPA (+)
	1	216011212	216011614	*USH2A*	47	0.47	MLPA (+)
	1	216017384	216018005	*USH2A*	46	0.48	MLPA (+)
	1	216019005	216019497	*USH2A*	45	0.46	MLPA (+)
RP-0830	12	88471664	88471865	*CEP290*	40	1.24	aCGH (-)
	12	88522578	88523012	*CEP290*	11	1.25	aCGH (-)
