# Supplementary material for: Panel-based NGS Reveals Novel Pathogenic Mutations in Autosomal Recessive Retinitis Pigmentosa
Source: Sci Rep. 2016 Jan 25;6:19531. doi: 10.1038/srep19531 (PMC4726392; doi:10.1038/srep19531)
Supplement: Supplementary Table 4 [file srep19531-s4.pdf]

Panel-based NGS Reveals Novel Pathogenic Mutations in Autosomal Recessive Retinitis Pigmentosa

Raquel Perez-Carro[1,2], Marta Corton[1,2], Iker Sánchez-Navarro[1,2], Olga Zurita[1,2], Noelia Sanchez-Bolivar[1,2], Rocío Sánchez-Alcudia[1,2], Stefan H. Lelieveld[3], Elena Aller[2,4], Miguel Angel Lopez-Martinez[1,2], Mª Isabel López-Molina[5], Patricia Fernandez-San Jose[1,2], Fiona Blanco-Kelly[1,2], Rosa Riveiro-Alvarez[1,2], Christian Gilissen[3], Jose M Millan[2,4], Almudena Avila-Fernandez[1,2,6], Carmen Ayuso* [1,2,6].

Supplementary Table 4. Novel missense variants predicted as pathogenic found in our cohort that were previoulsy identified in Spanish population.

Gene	Nucleotide change	Protein change	SIFT	Polyphen	Align GVGD	Mut.Taster	SPV
EYS	c.5510G>C	p.Trp1837Ser	Not scored	Pos.D 0.67)	Class C0	P	0.006
MPDZ	c.2344G>A	p.Gly782Arg	D(0)	Pr.D(1)	Class C65	D	0.015
